# Source of Knowledge Dynamics—Transition from High School to University

**DOI:** 10.3390/e22090918

**Published:** 2020-08-21

**Authors:** Shahrazad Hadad, Mihai Dinu, Robert Bumbac, Maria-Cristina Iorgulescu, Ramona Cantaragiu

**Affiliations:** UNESCO Dept. for Business Administration, The Bucharest University of Economic Studies, Calea Grivitei 2-2A, 010731 Bucharest, Romania; mihai.dinu@eam.ase.ro (M.D.); robert.bumbac@com.ase.ro (R.B.); cristina.iorgulescu@com.ase.ro (M.-C.I.); ramona_cantaragiu@yahoo.com (R.C.)

**Keywords:** source of knowledge, higher education, knowledge management, cognitive development in adolescence, long life learning, Markov chain, Likert scale, stationary distribution

## Abstract

The paper addresses the dynamics of education by using Markov chains, a powerful probabilistic model able to make predictions on how sources of knowledge either change or stabilize over adulthood. To this end, each student filled in a survey that rated, on a scale from 1 to 5, the utility of five different sources of knowledge. They completed this survey twice, once for their previous and once for their current education. The authors then fitted a Markov chain to these data—essentially, calculating transition probabilities from one ranking of sources of knowledge to another—and inferred the final maximum utility sources of knowledge via the stationary distribution. The overall conclusion is the following: even if the professor used to play a crucial role in early development, students have the tendency to become independent in their learning process, relying more on online materials and less on printed books and libraries.

## 1. Introduction

Education is a long debated topic and is perceived through positive, negative and neutral lenses. In order to alleviate the gloominess that has been surrounding education, the authors have decided to embark on a research meant to show how the sources of knowledge used by students change or remain unaltered once the subject transits from high school to university, advocating for the efficiency of education in the vein of knowledge management thought.

Some researchers in their study related to metacognition identified two categories of students: (a) those with lower level of metacognition whom were associated with a uni-dimensional measure and exhibited lower levels of deep learning strategies and performance and (b) those with higher levels of metacognition associated with a bi-dimensional measure of regulation and knowledge, whom manifested significantly higher utilization of deep learning strategies [1].

The education literature convincingly argues that students have different learning styles and use different tools. Most frequently reported, in business education at least, are the following: Professor’s lectures, books and libraries, case studies, success stories of business people, projects and seminars, online sources, interactions with colleagues [2,3]. Though Professors’ lectures are believed to be a form of organizing the educational process, in this paper they do not necessarily refer to the professors’ written handouts or form of organizing the course, but to the knowledge they convey to students. Books and libraries, despite the fact that books are means of learning, and libraries are dedicated spaces, also reflect on the knowledge students extract by interacting with them. On the one hand case studies represent a teaching method, on the other hand and in line with our research they stand for the knowledge they emanate after students interact with them. The online sources are a means of learning and, implicitly, a source of knowledge. The interaction with colleagues is first of all a form of communication in the educational process, and, secondly, very easily assimilated to shared dyads of metacognition in a collaborative setting [4]. Ultimately, co-regulation in collaborative learning has been rendered as a sustainable endeavor for the learning process and education ecosystem [5].

After carefully evaluating the theoretical framework, we decided to categorize Interaction with colleagues also as a source of knowledge, since we do not refer to a mere physical interaction/communication, but to the actual exchange of information and transfer of knowledge.

As the preference is influenced by the cognitive development of the subjects (which is obviously dependent on time), one can simplify the dynamics of the process down to a two-iteration conditioning, leading to a Markov chain model. The stationary distribution of this Markov chain is translated into a preference vector over the sources of knowledge in our study.

Using a 10-item questionnaire addressed to a large group of bachelor students from a Business and Economics university, the utility of five sources of knowledge—previously identified and confirmed by literature—was rated on a five-point Likert scale. Actually, the survey consisted of five different questions that have been answered twice: once for their previous and once for their current education approach.

To our knowledge there is no similar research in the education literature, assuming a Markov dependency among the students’ preferences towards various sources of knowledge. There is no guarantee that education preferences are described by such stochastic models, and no guarantee either that all students share the same Markov chain. Yet, this is always the trade-off between mathematical modeling and practice, especially within the realm of social sciences.

The current research does not resemble any of the Markov models presented in the literature for academic dynamics. Previous research employs data from the administrative level within university, referring to inbound and outbound mobility of students. By contrast, we gather primary data directly from students, by means of a questionnaire. Subjects rate their preference with respect to sources of knowledge and support actions, at two different moments in time.

By firstly treating each source of knowledge separately, and then by looking at all five together—which required an original, carefully constructed dichotomization algorithm for the Likert scale—we fitted six different Markov chains to the survey data. As a result, several probability distributions were computed and analyzed:

- the two static distributions corresponding to high school and university
- the five limit/stationary distributions corresponding to the individual source of knowledge
- the stationary distribution among the five sources of knowledge.

We consider the last distribution of paramount interest, as rendering the adult’s preference towards the five sources of knowledge, providing a clear indication on how long-life-learning programs should be designed. Unsurprisingly, results show that professors, printed books and libraries do not play a crucial role anymore, as students become more and more independent and online learning becomes predominant.

As portability of psychometric instruments between different cultures, economies and continents is usually limited, see e.g., [6,7], the authors do not expect unbounded generality for the conclusions of their analysis.

Section 2 presents the current state in learning theory and the respective Markov models and Section 3 introduces two particular dichotomization methods employed for the analysis of the questionnaire. Section 4 depicts the Markov transition matrices obtained under various partial and global perspectives, together with the two stationary distributions obtained under the two different dichotomization methods, while Section 5 and Section 6 conclude the study and point out directions for future research.

## 2. Literature Review

Education has evolved at the same pace with the technological advancements. If, at the beginning it simply consisted in a mere dyad of the professor and the student, nowadays we are talking about an intricate ecosystem that embodies both parties alongside different sources of knowledge, topped by the computer-assisted learning, perceived as a reliable and effective tool for helping people learn in an orderly and customized manner [8]. Although different experiments delivered inconclusive results on this source of knowledge [9,10,11], we attribute the counter results to the fact that technology in education has not yet been established for such a long period of time as for us to be able to study its effects over the long run. In particular, business education should adapt its design to the complexity of the real business environment dynamics and to the need of strategic thinking. As such we posit that among sources of knowledge, the business knowledge ecosystem should include the professor, study cases, online business sources, student projects, practical business activities, success stories of business people, Erasmus and other international projects [2].

Markov chains and learning theory have a long history together, going back in time as far as the 1950s [12,13]. For several decades literature has been confined to scholar applications only, seeing learning as a stochastic process based on responses to psychological stimuli under reinforcement through penalties and rewards, see e.g., the excellent monograph [14]. The advantages of such carefully constructed theoretical models consist in analytic formulae of the stationary distribution, useful in case of a large state space. Yet, finding an intrinsic relationship among states leading to a close-form stationary distribution is always a tricky, highly unlikely mathematical event, as witnessed by the Markov chain analysis of how opinion changes within a community. The Markov chain was built by considering a lattice-like spatial representation of the population; the opinion of each person is affected (probabilistically) only by a small group of peers in her/his neighborhood [15,16,17,18,19].

Apparently, the topic of the present paper—how students shift from one source of knowledge to another—could also be read as change of opinion paradigm. We think this is not the case, because learning works on a deeper, more intrinsic psychological level than a simple change of opinion. That is the reason for building our Markov chain from empirical data, instead of considering an established theoretical model. This also corresponds to the more recent trend in literature, where accent shifts onto practical oriented studies, ranging from modeling faculty movement inbound and outbound higher education institutions [20], to describing student flow throughout: different terms, or one or more degree programs in civil and military academia [21,22,23].

One paper analyzes how students’ achievements during the semester affect the final outcome of the exam [24]. This is a typical example of reducible Markov chain, with the final grade seen as the absorbing state that cannot be anymore transitioned from. Academic performance in a Slovenian higher education institution is modeled by an absorbing Markov chain in [25]. Five transient and two absorbing states are identified, then students’ progression towards the next stage of the educational program is estimated in terms of expected duration of the study. An extended Markov model of the same type is used for the movement of undergraduates through the higher education system in Australia - with 51 transient and two absorbing states [26]. Given the age of student when they commence a course, the model estimates both the probability, and expected time for their completing the course. Similar studies inferring reducible/absorbing Markov models from statistical data on student flow in universities from India, Saudi Arabia and US can be found in [27,28,29].

As already stated, the present study does not resemble any of the previously discussed Markov models for student movement inbound and outbound of the university. Instead of sampling data from faculty (administrative) level, our analysis is based on a questionnaire, asking each student to rate their preferences towards a set of sources of knowledge at two distinct moments in their scholarly life. The sampling method is different and the results are different as well: instead of reducible Markov models exhibiting one or more absorbing states, the Markov chain we obtain is ergodic, with a proper stationary distribution, able to predict the subject’s preference towards the sources of knowledge during adulthood.

Another fast growing direction in education literature taking into account the temporal and sequential nature of the learning process is the socially- and self-regulation learning (S-SRL) [30]. Developed within the larger context of process mining, which aims at identifying educational process models based on event data [31], S-SRL builds on the idea that learning unfolds in three main phases (preparation, execution and reflection) among which there is a cyclical relation. Yet, the authors do not proceed further to induce a Markov chain structure among the three states (phases). Another work in the S-SRL framework analyzes the student restudy decisions based on a statistical factor model, with clusters distinguishing between known and unknown items [32]. Again, that is a static structure which does not capture the dynamics of the learning process. It looks like the entire S-SRL literature considers the existent mathematical formalism to be “deterministic in nature, lacking the means to state that a transition from one state to the next will take place (only) with some probability” [33]. Yet this is exactly the finite homogeneous Markov chain paradigm we apply in this paper.

We cannot simply conclude based on a bird’s eye view on Markov chains and learning without pointing out a spectacular literature which completely reverses the research paradigm. Instead of imposing an external stochastic model on students behaviour, some authors build their Markov model on the idea that the human brain itself works by recovering a probabilistic process from a sample of empirical data [34]. According to this analysis, the brain has intrinsic, native probabilistic structures which—exactly like the pre-defined grammatical rules—operate unconsciously in the process of inferential thinking [35,36].

## 3. Materials and Methods

### 3.1. Data

We designed a questionnaire which was physically distributed to 436 undergraduates affiliated to a business administration faculty. After a thorough pre-analysis data screening of the returned questionnaires, a remainder of 269 questionnaires were evaluated as valid. Under these circumstances, the obtained response rate was 61.69%. For a confidence level of 95% and a confidence interval of 4%, for a population of 485, the recommended sample size was 269. The average age of the sample is 20, the gender distribution is 60% female, 40% male and the year distribution is 47% second year and 53% third year since we figured that for first year students it would be rather difficult to assess the impact of specific sources of knowledge.

The questionnaire attempted to decrypt the dynamics regarding their sources of knowledge preference. The answers to each question are registered on a typical 1 to 5 Likert scale, then translated into an Excel database in order to build the Markov models.

We applied a filtering procedure to the database of 269 respondents, by excluding subjects that responded 1 (never) to all five questions of a set—either with respect to current or previous use of sources of knowledge. The motivation is straightforward: a response of all 1’s means either that (a) the subject has no propensity for academia, or (b) the factorization of the education process as provided by Table 1 is not representative at all. Fortunately, only one of the 269 subjects had an all 1s line of response, therefore alternative (b) is not sustained. By canceling out that one respondent, we were left with a database of N=268 valid answers.

### 3.2. Markov Chains

A stochastic process is a sequence of random variables with an intrinsic relationship among the variables. The finite homogeneous Markov chain is the classical example, defined on a finite set of states S={1,2,…,n}, with transition probabilities from one state to another depending only on the current state. We denote the transition probability from state *i* to state *j* by $p_{ij}$, and gather all these probabilities into a square non-negative transition matrix $P={\left(p_{ij}\right)}_{i,j=1,n}$. Since *P* does not change over time, the Markov chain is called homogeneous. Considering a fixed row *i*, probabilities $p_{ij}$ sum to one, and thus, for each state *i*, transition from state *i* to the next one defines a random variable. A matrix *P* with this property is called stochastic. As the sequential movement of the chain inside the set *S* continues for infinitely many steps, *P* governs the short and long term dynamics of the process. What makes Markov chains so attractive, both to theoreticians and practitioners, is the limit property of the powers ${\left\{P^{t}\right\}}_{t\to \infty }$. Under non-restrictive conditions—*P* positive, e.g.,—the power sequence converges to a matrix with identical rows, the generic row being the so-called stationary distribution, often seen as a predictor for the long-term behaviour of the process (Notice that, if *P* depicts the transition probabilities in one step, $P^{t}$ depicts transition probabilities in *t* steps.). For a rigorous introduction of the concepts and main results on Markov chains the reader is referred to some important monographs [14,37,38].

In case the Markov chain matrix is not provided by the theory, it is built from empirical data. One starts with *N* observed transitions among the states in *S*, represented by positive integers $N_{ij}$, such that $\sum_{i,j=1}^{n}N_{ij}=N$. Then for any pair (i,j) the Maximum Likelihood estimate of transition probability $p_{ij}$ is given by the relative frequency [39,40]

(1) $$p_{ij}=\left\{\begin{array}{l} N_{i,j}/\sum_{j=1}^{n}N_{i,j}\\ 1/n\;,\;\mathrm{if}\;\mathrm{the}\;\mathrm{above}\;\mathrm{denominator}\;\mathrm{is}\;0\;. \end{array}\right.$$

There are two steps in applying Markov chains for modeling a real-life situation. First, one needs to build empirically the transition matrix *P*, describing the short term behaviour of the process in study. Second, depending on the particular transition matrix obtained, information on the long term behaviour of the process can be obtained by multiplying *P* by itself and looking at the asymptotics of the powers $P^{t}$—referred to in literature as the power method. Alternatively, the stationary distribution can also be obtained algebraically, using the eigenvalue–eigenvector decomposition method [14].

### 3.3. Building the Models

We considered five sources of knowledge, designed to synthesize, from a knowledge management perspective, the main factors that contribute to the education process, at an academic level, as depicted in Table 1 [2,3].

With respect to each of the five sources, students were required to rate, on a scale from 1 to 5, the degree (1 = never, 2 = seldom, 3 = sometimes, 4 = often, 5 = very often) to which they use/have used the respective source of knowledge in their *current/previous* education program (bachelor studies/high school). This makes the process associated to each particular source of knowledge ideal for the application of Markov chain theory, by defining the states as the ordinal Likert values ranging from 1 to 5.

We counted how many transitions we have from 1 to 1, from 1 to 2, and so on, gathered all these values in a 5×5 integer (transition frequency) matrix, which we normalized into a stochastic matrix by dividing each component with the sum of the corresponding row. In this manner we obtained five different Markov chains, one for each source of knowledge, each being defined by a distinct transition matrix. All these models pertain to the reducible case, with absorbing states at the extreme points of the Likert scale.

At a first glance, the five Markov chains seem to fulfill the aim of our quantitative analysis, which was inferring long-term behaviour from short-term transitions. Yet, one should notice the hidden assumption in the previous modeling: the rankings for each source of knowledge are evolving independently. However, it is more likely that the evolution of one source of knowledge was coupled with the evolution of another source of knowledge.

As a consequence, we opted for designing a more elaborate Markov chain, that accommodates transitions among different sources of knowledge. To this end, the 5-point Likert scale had to first be dichotomized, as we needed to extract, for each respondent, the source(s) of knowledge that was (were) dominant for either high school or university. We claimed that this new Markov model has the potential of revealing the true cognitive development during adolescence, as students move from one education program to another.

### 3.4. Dichotomization

In order to build transition probabilities among sources of knowledge, one should define the state space as the set of all possible rankings occurring in the survey, then infer transition probabilities. However, the computational complexity is too large for that to work: the state space would be $5^{5}=3125$, yielding a sparse transition matrix of size $5^{10}$. Under these conditions, we downsized the original state space to more manageable dimensions.

Such a procedure is known in literature as dichotomization, and is usually applied to transform the ordinal Likert scale into a binary one - that is, to transform the original 1–5 answers of the subjects into nominal *yes/no* replies. Although the methodological literature in psychology and social science argues strongly against dichotomization, we believe this is a rare occasion when such a procedure is justified [41]. Notice that the procedure used in our paper was not the usual dichotomization, in the sense that it did not impose any cut-value α between 1 and 5 and treated all values below α as zero and all values above α as one.

Instead, we compressed the state of all five Likert rankings into a list of top-ranked sources. For instance, if a student scored (2,1,3,3,2) on the set of sources of knowledge (A,B,C,D,E) in Table 1, we registered this entry under the set of maximal positions *C* and *D*. The actual value of the maximum, which in our case equalled 3, was discarded. So, instead of looking at all the possible numerical combinations of the 1-5 rankings, we only looked at the subsets of {A,B,C,D,E}. As a result, the state space dimension was downsized to $2^{5}-1=31$ (where the empty set was excluded), and the transition matrix was also downsized to $31^{2}$. The dichotomization procedure is contained in Steps 1,2 from the algorithm below.

### 3.5. Global Markov Chain

Step 1 We initialized a 31×31 frequency matrix *F* with zero, which gathered all transitions among previous–current states {A,B,C,D,E}. To improve readability, we assumed the rows and columns of this matrix have non-numerical indices, namely the 31 subsets of {a,b,c,d,e}.
Step 2 To fill in the positions in *F*, for each respondent *i* (i=1,N) we checked the five questions on previously used sources of knowledge, found the maximal value of their responses and extracted the item(s) for which the maximum was attained; we did the same for the five questions on currently used sources of knowledge. Then we added 1 to the position $F_{i,j}$ in *F* that corresponded to maximal response value in the previous use (row index) *i* and to maximal response value in the current use (column index) *j*. For example, if one student responded (2,1,3,3,2)−(3,1,2,5,2) we added 1 on position $F_{cd,d}$ (In a simple, permutation like codification, this corresponded to i=13 and j=4, so we added 1 to $F_{13,4}$ in this case.).
Step 3 We transformed frequency matrix *F* into a stochastic matrix *P* of the same size: on each row, we divided each component by the overall sum of the row.
Step 4 We multiplied *P* by itself until it stabilized (To speed things up, we computed only the powers $P^{2}$, $P^{4}$, $P^{8}$ etc.). Termination criterion: the maximal difference between corresponding components in any two successive rows in $P^{t}$ less than $10^{-4}$.
Step 5 The identical row from the stabilized matrix $P^{t}$ is the stationary distribution of the 31-state Markov chain, call it *p*. In order to transform *p* into a 5-length probability vector π, we re-distributed the probabilities uniformly. For example, if p(cd)=0.3 we charged 0.3/2 on both π(c) and π(d).
Step 6 π was the stationary distribution over the five sources of knowledge.

## 4. Results

The current research yielded two major results. The first one would be that the evolution of the five sources of knowledge under scrutiny was coupled, which derived from the statistical analysis of correlations. The second conclusion refers to the particular sources of knowledge that would be eventually favored by the students. This is explained at length in the following.

Firstly we built five individual matrices corresponding to each source of knowledge, namely *Professor*, *books*, *case studies*, *online* and *colleagues*. In each case, the states corresponded to the ordinal numbers 1–5 of the Likert scale, while the (i,j) matrix component is the transition probability in one step from state *i* to state *j*. Since each of the matrices contained at least one component equal to 1, they all fell into the reducible case, leading to Markov chains that converged to the respective absorbing states. The five stationary distributions are depicted in Table 2. Note that in case of absorbing chains we did not have proper stationary distributions, the limit being dependent on the initial state.

After screening the results, the conclusion was that the first two sources of knowledge, Professors and books, were prevalent during the long-time development of the cognitive process, since both exhibited a single absorbing state, at the maximal value of the Likert scale. As for the other three sources of knowledge, the limit matrices were not conclusive, each exhibiting two absorbing states, at both extreme values of the Likert scale.

Despite these results, analyzing the five sources of knowledge separately was a misleading endeavor, as it assumed items were statistically independent. This assumption was contradicted by the SPSS analysis of the Pearson correlations provided in Table 3 and Table 4, for both high-school and university programs.

**Figure 1:**
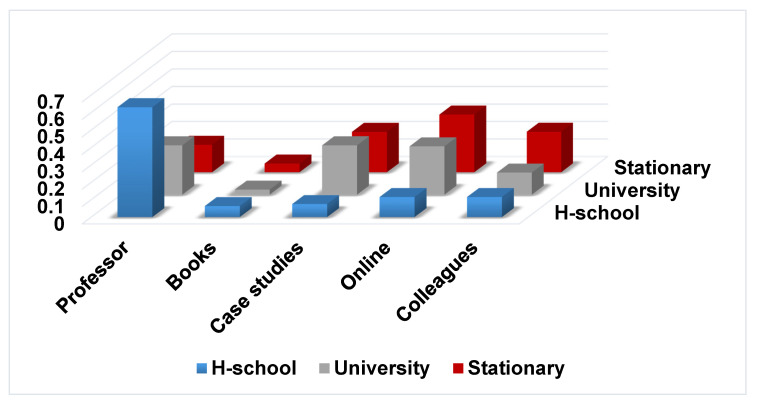
Probability distributions: static versus stationary.

Next, we used the Global Markov chain algorithm from Section 3.5 to build a new transition matrix. For every respondent and each moment in time, previous and current, we transformed the actual reply of a subject into a maximum-index vector, from which we built the frequency matrix *F*, with positive integers, over the 31 states of the Markov model. Matrix *F* was easily transformed into a stochastic transition matrix *P*, which was multiplied by itself until all the rows converged to a unique probability vector. This limit row was the stationary distribution π, depicted in Table 5 together with the static distributions computed for *High school* and *University*. A graphical representation is provided in Figure 1.

As the stationary distribution captured the long-term, steady-state preference distribution over the five sources of knowledge of an adult, graduate of Business and Economics, during her/his future professional years, the interpretation is straightforward: The online sources were mostly engaged, interaction with colleagues, and case studies and seminars came second, lectures given by professors/experts were not that used anymore, while printed books and libraries became less used.

The analysis of the stationary distribution also supported the decision of grouping the five sources of knowledge into a single Markov chain. As the first two sources of knowledge scored high (Likert value 5) in Table 1, one would expect high probabilities on the first two components of π in Table 5. On the contrary, π charging lowest on the first two positions argued against de-coupling the Markov models.

## 5. Discussion

The paper presents a stochastic model of the way in which learning transitions among different sources of knowledge while subjects move from high school to university. Education is a complex ecosystem relying on dedicated means. Literature on this matter is extensive and has filtered as most important sources the following: Professor, case studies, online sources, student projects, practical activities and peer-to-peer teaching and learning.

The previous Markov models within the academic spectrum were devoted only to inbound-outbound student flows, as measured by data collected at faculty level. To the extent of our research, we could not find studies addressing the dynamics of students’ cognitive development, which is an ever changing process that can be expressed by the preference of the subject towards one or other source of knowledge, in accordance to their brain dominance. Inspired by the knowledge management paradigm, we identified five sources of knowledge that we consider common for most education programs: Professor’s lectures, books and library, case studies and seminars, online sources, and interactions with colleagues.

A questionnaire addressed to students from Business and Economics bachelor studies required them to rate their preference with respect to each of the five sources of knowledge, at two different points in time: high-school and university. Responses were measured on a five-point ordinal Likert scale. Based on the two-stage time conditioning, five probability transition matrices were constructed (one for each source of knowledge). Performing de-coupled Markov analyses for each source can be a flawed endeavor, since it is more reasonable to assume that the learning process is a whole, with all five sources of knowledge evolving together as a mechanism.

The new model required first a dichotomization of the Likert scale, leading to a different, extended Markov matrix, aggregating transition probabilities among the sources of knowledge themselves. The latter transition matrix is primitive and characterized by a stationary distribution which captures the limit behaviour of the entire cognitive process. In a straightforward reading, the stationary distribution is a predictor for the individual preference among the sources of knowledge during adult life.

From the stationary distribution of this transition matrix, one may infer which mode of learning is the most useful in the long run. This approach can help customize the long-life learning programs designed by different institutions or policy makers. According to our results, the extreme points on the utility scale are: online sources being awarded a maximum, with 38%, while the minimum is scored by books and library, with a mere 6%. This re-acknowledges that the most representative characteristic of the Z generation the technological savviness. Between these two extremes, case studies and seminars and interactions with colleagues score equally moderate (at around 20%), while Professor’s lectures may also be seen as declining (12%).

## 6. Conclusions

The contribution of the current paper is twofold: the first one is represented by the proved dependency among the five sources of knowledge which are the subject of study; the second one refers to the particular sources of knowledge that would be favored during adulthood.

Since there are no similar Markov models of source of knowledge dynamics in literature, likewise comparison cannot be delivered or assessed. In this vein, an important line of future research consists in validating the stationary distribution obtained in the present work by compiling a similar questionnaire to either master or PhD students.

In this scenario, we could consider more than two moments in time for the construction of the Markov model—e.g., should the respondents be PhD students, the reference moments would be the following, in an ascending order: high school, bachelor, master, and PhD. This would imply either of the following two paths: (a) a similar two-stage Markov chain, but with the corresponding matrix averaged along all the three consecutive transitions: high school–bachelor, bachelor–master, and master–PhD; (b) an extended, four-step Markov chain, accounting for a higher degree of dependency (that is, the current state is not conditioned only by its previous, but also by its previous to previous, and so on).

We acknowledge the limitation of the current study with respect to evaluating a previous moment in time, based on subjective recollection. This limitation could be addressed by conducting a more elaborated longitudinal study, over several years, and tracking the same subjects throughout a couple of academic years. Consequently, though a limitation, this also constitutes a pertinent line of future research.

One limitation, to the common 1 to 5 Likert scale used in the paper, is that different students have different definitions of what a particular numerical label means. To test the reliability of the results, a possible research avenue consists in undertaking a similar Markov research, however using different scales, such as: Saaty scale, Ma-Zheng scale and even the linguistic scale, and perform a comparison between the results. In such case, this will give rise to a new dichotomization algorithm, particularly tailored to the specificities of the newly defined scale.

According to constructivism theory pertaining to social sciences, we cannot claim generality for the results. Still, our deduction is consistent with the new globalization and virtual era paradigm characterizing the 21st century. Alongside the principles of fair-play in research, other authors can replicate the same questionnaire with students from other countries, and perform the same Markov analysis. The stationary distribution may look differently. On the other hand, since the respondents of the present survey belonged to an Economics and Business university, it would be interesting to test whether results change dramatically if considering a sample from a different academic domain.

## Figures and Tables

**Table 1:** Sources of knowledge.

A	*Professor*’s lectures
B	Physical *Books* and library
C	*Case studies* and seminars
D	*Online* sources
E	Interactions with *Colleagues*

**Table 2:** Stationary distributions on individual items.

ItemScore	1	2	3	4	5
**Professor**	0	0	0	0	1
**Books**	0	0	0	0	1
**Case studies**	1/0	0	0	0	0/1
**Online**	1/0	0	0	0	0/1
**Colleagues**	1/0	0	0	0	0/1

**Table 3:** Correlations between individual items—high-school.

	A	B	C	D	E
**A**	1	0.094	0.191 *	–0.207 *	0.040
Sig.		0.126	0.002	0.001	0.513
**B**	0.094	1	0.213 *	0.055	0.005
Sig.	0.126		0.000	0.371	0.933
**C**	0.191 *	0.213 *	1	0.248 *	0.303 *
Sig.	0.002	0.000		0.000	0.000
**D**	–0.207 *	0.055	0.248 *	1	0.270 *
Sig.	0.001	0.371	0.000		0.000
**E**	0.040	0.005	0.303 *	0.270 *	1
Sig.	0.513	0.933	0.000	0.000	

* Significant at 0.01 (2-tailed). N = 268.

**Table 4:** Correlations between individual items—university.

	A	B	C	D	E
**A**	1	0.208 *	0.195 *	–0.054	0.157 *
Sig.		0.001	0.001	0.381	0.010
**B**	0.208 *	1	0.163*	–0.119	0.247 *
Sig.	0.001		0.007	0.051	0.000
**C**	0.195 *	0.163 *	1	0.018	0.271 *
Sig.	0.001	0.007		0.763	0.000
**D**	–0.054	–0.119	0.018	1	0.105
Sig.	0.381	0.051	0.763		0.086
**E**	0.157 *	0.247 *	0.271 *	0.105	1
Sig.	0.010	0.000	0.000	0.086	

* Significant at 0.01 (2-tailed). N = 268.

**Table 5:** Static and stationary (π) distributions for sources of knowledge.

	Professor	Books	Case studies	Online	Colleagues
High school	0.63	0.06	0.07	0.12	0.12
University	0.28	0.03	0.28	0.28	0.13
Stationary	0.12	0.06	0.21	0.38	0.23
